# Validation of factor structure of the neurodevelopmental parent report for outcome monitoring in down syndrome: confirmatory factor analysis

**DOI:** 10.3389/fpsyt.2024.1293937

**Published:** 2024-03-05

**Authors:** Nicole T. Baumer, Katherine G. Pawlowski, Bo Zhang, Georgios Sideridis

**Affiliations:** ^1^ Division of Developmental Medicine, Boston Children’s Hospital, Boston, MA, United States; ^2^ Department of Neurology, Boston Children’s Hospital, Boston, MA, United States; ^3^ Harvard Medical School, Harvard University, Boston, MA, United States; ^4^ Biostatistics and Research Design Center, Institutional Centers for Clinical and Translational Research, Boston Children’s Hospital, Boston, MA, United States

**Keywords:** down syndrome, autism spectrum disorder, parent reported outcome measure, ASD-PROM, ND-PROM, confirmatory factor analysis, developmental questionnaire

## Abstract

**Introduction:**

The Neurodevelopmental Parent Report for Outcome Monitoring (ND-PROM), initially developed to monitor developmental and behavioral functions in children with autism spectrum disorder (ASD), assesses symptoms across a wide range of domains relevant in Down syndrome (DS).

**Methods:**

Psychometric properties of ND-PROM were assessed in 385 individuals with DS and 52 with a combined diagnosis of DS and ASD (DS+ASD), whose caregivers completed the ND-PROM questionnaire for a clinical visit in a specialized Down syndrome program at a tertiary pediatric hospital. Confirmatory factor analysis was conducted to evaluate the internal structure validity of the ND-PROM. Measurement invariance was assessed, with a comparison group of 246 individuals with ASD, and latent mean differences between the DS and ASD-only groups, as well as the combined DS+ASD groups, were assessed.

**Results:**

Findings support the existence of the 12 clinically-derived factors in the DS population: Expressive Language, Receptive Language, Adaptive skills/Toileting, Social Emotional Understanding, Social Interaction, Independent Play, Sensory Processes, Challenging Behaviors, Impulse/ADHD, and Mental Health. Differences in response patterns of development and behaviors were observed between those with DS and those with ASD, including those with DS having higher abilities in nonverbal communication, social emotional understanding, and social interaction, and fewer restricted and repetitive behaviors and interests, impulsivity or ADHD symptoms, and mental health concerns compared to those with ASD. Individuals in the DS+ASD group had more difficulties with expressive and receptive language, nonverbal and social communication, social interaction, independent play, and adaptive skills than either the DS-only group or the ASD-only groups.

**Discussion:**

The ND-PROM has a desirable factor structure and is a valid and clinically useful tool that captures a range of distinct and independent areas of developmental and behavioral functioning in DS, for individuals with and without an ASD diagnosis.

## 1 Introduction

Down syndrome (DS), caused by the presence of all or part of an extra chromosome 21, occurs in about 1/700 births (1). DS is the most common genetic cause of intellectual disability (ID), though cognitive, language, and adaptive abilities vary greatly (2–7). DS is associated with a high prevalence of co-occurring neurodevelopmental, behavioral, and mental health conditions that can greatly impact overall functioning (8). Co-occurring ASD is particularly prevalent, occurring in up to 39% (9–11), and is typically associated with lower cognitive and language abilities, and higher rates of behavior problems (12). There is also an increasingly recognized phenomenon of unexplained regression in DS, now known as Down Syndrome Regression Disorder, which is associated with loss of skills and onset of autistic-like behaviors or catatonia (13–15). Thus, clinical care for individuals with DS requires clinicians to monitor developmental and behavioral progress across multiple domains, and to identify and manage any unexpected changes in behavior or deviations in development.

In order to enable efficient, patient-centered care, a standardized approach is needed that tracks a wide range of potential symptoms and identifies those at a heightened risk for co-occurring neurodevelopmental or mental health conditions. Parent-reported measures have been used in DS, including the Aberrant Behavior Checklist (12), Child Behavior Checklist (16), Social Responsiveness Scale (17), the Social Communication Questionnaire (18), and the Screen for Child Anxiety Related Disorders (19). However, many tools have not been validated in people with DS or are used only in specific age groups or to target particular symptom clusters. Evaluation of the breadth of symptoms and concerns that children and adolescents with DS may experience therefore necessitates the use of multiple scales. Thus, there remains a great need for a single tool that can be used to obtain information on developmental and behavioral domains applicable to people with DS, across a wide range of ages, developmental stages, and functional levels, that can ultimately be used to monitor clinically-relevant symptoms and skills.

The Neurodevelopmental Parent Report for Outcome Monitoring (ND-PROM; previously published as the Autism Spectrum Disorder Parent Report for Outcome Monitoring, ASD-PROM) is a freely-available tool initially developed to clinically monitor caregiver report of developmental and behavioral functions in children with ASD (20). The ND-PROM contains 128 Likert-scale items that cover a wide range of developmental skills and behaviors relevant to children with neurodevelopmental disabilities. Prior work using the ND-PROM in individuals ages 2-20 years old with ASD demonstrated clinical utility, test-re-test reliability, and good convergent validity with the Vineland-II (20). Further development of the ND-PROM involved delineation of 93 of the individual items into 12 clinical domains, which were subsequently supported using confirmatory factory analysis in the ASD population: Expressive Language, Receptive Language, Nonverbal Communication, Social Communication, Social Interaction, Independent Play, Adaptive Skills, Restricted and Repetitive Behaviors, Sensory Processes, Challenging/Aggressive Behaviors, Impulse/ADHD, and Mental Health (21). Items related to sleep, possible emergence of epilepsy, and drug or alcohol use from the original survey were not included in the factors. This suggested that the ND-PROM has good potential for independently assessing these key functional domains and identifying domains of relative strengths and weaknesses (e.g. restricted and repetitive behaviors and interests, or impulsivity or ADHD symptoms), which can identify targeted areas for intervention (21).

Given the applicability of these skill and behavioral areas in the clinical management and treatment of Down syndrome, the Boston Children’s Hospital Down Syndrome Program began implementation of the ND-PROM as part of standard clinical care to gather developmental and behavioral information from parents and caregivers about their children with DS prior to clinic visits. While previous confirmatory factor analysis in a population of children with ASD confirmed 12 separate clinical domains for which the questions were best represented (21), it is not clear that the same skills, symptoms and behaviors track similarly in a population of children with Down syndrome, which is a different neurodevelopmental condition with different neurocognitive and behavioral profiles. Therefore, the current study aims to assess psychometric properties of the ND-PROM in Down syndrome.

This paper describes the internal structure validity of the ND-PROM in a large clinical population of children and adolescents with DS, some of whom also have ASD (DS+ASD). Using Confirmatory Factor Analysis, we will examine factor structure and internal consistency of the measure in a new clinical population, determine the factor structure, confirm the measurement invariance of the ND-PROM, and assess latent mean differences among groups.

## 2 Methods

### 2.1 Participants and procedures

Participants included 385 individuals with DS and no diagnosis of ASD, along with 52 individuals with a dual diagnosis of DS and ASD (DS+ASD) who were seen in a specialized Down Syndrome Program in a tertiary pediatric hospital from 2017-2021. Patients were assessed and followed by specialty providers who regularly evaluate for ASD as part of their clinical practice using Diagnostic and Statistical Manual, 5^th^ Edition (DSM-5) criteria (22). A comparison group consisting of 246 patients with a diagnosis of ASD and not DS were used to test measurement invariance, and to assess latent mean differences among DS, ASD, and DS+ASD groups. Details about this group, including diagnostic determination and data collection methodology, is available in Levin et al. (20). Caregivers completed the ND-PROM as part of standardized clinical procedures prior to clinical visits, to streamline clinical history taking and developmental and behavioral monitoring as a quality improvement initiative in the programs. Participants completed the ND-PROM using either a web-based system in which parents received automated prescheduled emails with secure links to complete the questionnaire online, or by completing a PDF or paper copy of the ND-PROM. The study was approved by the Boston Children’s Hospital Institutional Review Board.

### 2.2 Data analyses

All analyses were conducted using Mplus 8.9 (23). The level of significance was set to 5% for a two-tailed test.

#### 2.2.1 Internal consistency

In order to measure internal consistency, which allows us to examine how reliably the ND-PROM is able to address the constructs it is meant to measure, two coefficients were engaged: the popular alpha of Cronbach and McDonald’s omega (24–26), which is appropriate for non-tau equivalent instruments, where each factor is not assumed to have equal item-latent variable relations.

#### 2.2.2 Confirmatory factor analysis to examine internal factor structure

Internal factor structure was examined using the Confirmatory Factor Analysis (CFA) framework using the weighted least squares estimate using mean and variance adjustments for the expected non-normality of ordinal indicators. A 12-factor CFA model for ordered categorical indicators using the Weighted Least Squares Mean and Variance Adjusted Estimator (WLSMV) was estimated assuming the presence of 12 distinct, correlated dimensions. This covariance modeling approach estimates discrepancies between the population variance-covariance matrix and the sample-based matrix. Model fit is assessed using both absolute and relative criteria of exact fit, close fit, or not so close fit (27). Exact fit is based on the assumption that there is no discrepancy between hypothesized and estimated variance-covariance matrices, Σ(θ). It represents an extremely strict set of evaluative criteria in the measurement of real-life phenomena, as minimal discrepancies would result in large chi-square values, particularly in the presence of excessive power. Thus, evaluation of the magnitude of the chi-square values should be the last resort in evaluating model fit. The same logic applies also to evaluating residuals with RMSEA values equal to zero. Using values of the root mean square error of approximation (RMSEA) between 0.05 and 0.08 are suggestive of acceptable but still “not exact” fit.

In addition to relying on the RMSEA, a series of descriptive fit indices (28), relative or incremental (e.g., comparative fit index (CFI); Tucker-Lewis index (TLI)) were employed based on the discrepancy function, adjusting for model complexity (i.e., number of estimated parameters and degrees of freedom). A large number of simulation studies examining their strengths and weaknesses (29) have favored the CFI and TLI as being relatively unaffected by sample sizes and model complexity (30) and were thus, used in the present study. Evaluative criteria of proper model fit usually involve values greater than 0.900 or more recently greater than 0.950 on the descriptive fit indices (31), RMSEA values between 0.05 and 0.08 (i.e., between close and not-so-close fit), and non-significant chi-square values (a strict omnibus criterion).

#### 2.2.3 Measurement invariance

Among the available restrictive models to test for measurement invariance, typically three levels of restriction are utilized (32). These are termed configural, metric, and scalar, and contain the necessary restrictions to conduct test of significance at the latent means level, presuming they are all satisfied. Consequently, across the two populations of DS and ASD, the three levels were examined with the configural model testing the equivalence of the factor model’s simple structure, the metric model imposing the equivalence of factor loadings linking the items to the latent construct, and last, the scalar or “strong invariance” model testing, in addition to the factor loadings, the equivalence of the intercept terms (or thresholds in categorical indicators). Tests of significance by use of difference chi-square tests for nested models are constructed to test whether the additional constraints are justified.

In cases where the classic protocol of strong measurement invariance was not met, we deferred to the alignment procedure developed by Muthen & Asparouhov (33). This methodology utilizes the configural model of no invariance and identifies the largest number of invariant parameters by allowing factor means and variances to vary freely across groups. The model utilizes a simplicity function to identify as many approximately invariant parameters as possible with few non-invariant parameters, thus attaining the goal of validly comparing latent means and variances between groups. To conclude the presence of measurement invariance, the number of significant between group parameters needs to be minimal or less than 25%. If measurement invariance is achieved, a latent means comparison can be conducted between the DS, ASD, and combined DS+ASD groups.

#### 2.2.4 Effect size indicator

When latent means were contrasted, latent Cohen’s d effect size statistic was utilized which presents results in the standard deviation metric. Conventions for effect size are small (SD=0.2), medium (SD=0.5) and large (SD=0.8) (34). Meaningful differences are considered as those in excess of a 0.5 standard deviation.

## 3 Results

### 3.1 Sociodemographics

There were 204 (53%) males and 181 (47%) females in the DS group, 205 (83.3%) males and 41(16.7%) females in the ASD group, and 39 males (73.6%) and 14 females (26.4%) in the DS+ASD group (**Table 1**), with the chi-square test of the differences in proportions being significant [χ^2^(1)=62.828, *p*<0.001]. Females were significantly less represented in the ASD group and the DS+ASD group compared to the DS group alone. Mean age in the DS group was 9.22 years (SD=4.97), in the ASD group 9.03 years (SD=3.96) and in the DS+ASD group 9.66 years (SD=4.87) pointing to non-significant between groups differences [F(2, 681)=0.432, *p*=0.650].

**Table 1:** Demographic characteristics of study participants.

	ASD Participants n=246	DS Participants n=385	DS+ASD (n=53)
Age, median (IQR)	8.8 (6.4-11.9)	8.28 (4.9-12.3)	9.3 (6.3-13.0)
Sex, n (%)			
Male	205 (83.3%)	204 (53.0%)	39 (73.6%)
Female	41 (16.7%)	181 (47.0%)	14 (26.4%)
Race, n (%)	(n=200)	(n=385)	(n=53)
American Indian/Alaska Native	1 (0.6%)	0 (0%)	0 (0%)
Asian	9 (5.2%)	11 (2.9%)	2 (3.8%)
Black/African American	9 (5.2%)	21 (5.5%)	3 (5.7%)
White	133 (77.3%)	281 (73.0%)	38 (71.7%)
Other	20 (11.6%)	32 (8.3%)	7 (13.2%)
Unknown/Not Reported*	28 (6.4%)	40 (10.4%)	3 (5.7%)
Responder Education, n (%)			
Did not complete college	22 (12.4%)	71 (18.4%)	14 (26.4%)
Completed college or above	92 (50.8%)	212 (55.1%)	29 (54.7%)
Unknown/Not Reported	67 (37%)	101 (26.2%)	10 (18.9%)
Primary Communication Type Median age (IQR)			
Spoken Language	9.39 (7.82-11.52) (n=211)	9.31 (6.42-12.90) (n=320)	10.75 (7.89-13.73) (n=20)
Sign Language	2.59 (2.30-5.57) (n=9)	3.82 (3.08-4.84) (n=48)	6.31 (3.77-7.30) (n=11)
Picture communication system (e.g. PECS)	5.81 (4.08-7.52) (n=12)	6.55 (4.59-6.72) (n=5)	9.37 (8.19-14.88) (n=7)
Electronic communication system (e.g. Dynavox, iPad)	9.16 (7.07-11.87) (n=6)	8.24 (4.32-8.28) (n=5)	11.33 (7.67-13.66) (n=9)
Missing/not reported (n (%))	0	7 (1.8%)	6 (11.3%)
Maximum Length of Communicative Units Median age (IQR)			
Does not yet communicate	5.62 (3.07-8.87) (n=10)	7.29 (2.70-11.25) (n=7)	(n=0)
Uses one word/picture/sign at a time	4.41 (3.70-7.38) (n=20)	3.64 (3.21-5.07) (n=13)	6.37 (5.07-7.66) (n=2)
Uses to words/pictures/signs at a time	5.68 (3.36-9.31) (n=14)	6.58 (4.69-9.96) (n=50)	10.64 (8.24-13.70) (n=7)
Uses three words/pictures/signs at a time	8.20 (6.23-10.97) (n=26)	8.71 (6.41-12.43) (n=108)	11.07 (10.85-12.36) (n=5)
Uses full sentences	9.60 (8.27-11.72) (n=176)	11.20 (8.15-13.43) (n=123)	12.12 (10.02-17.56) (n=5)
Missing/not reported (n (%))	0	84 (21.8%)	34 (64.2%)

Sociodemographic and responder education level for participants. Median and interquartile range (IQR) is shown for age, primary communication type, and maximum length of communicative units; all other factors are presented with n and percent reporting. Race, ethnicity, and responder education were taken from the medical record and were not available for all participants. *“Other” includes people who self-identified as Multiracial.

### 3.2 Construct validity and internal consistency reliability of ND-PROM in DS

**Table 2** contains information about Cronbach’s alpha and McDonald’s omega across domains and groups. Estimates in alpha ranged between 0.578 and 0.925 in the DS group, between 0.632 and 0.932 in the ASD group, and between 0.538 and 0.924 in the DS+ASD group. The CFA model posited 12 latent factors as with the original ND-PROM in the ASD population (Levin 2022 vs SDBP Abstract). Global fit as judged by the chi-square test was significant, likely reflecting excessive levels of power. Use of descriptive fit indices and residuals pointed to acceptable model fit [CFI=0.90; TLI=0.90, RMSEA=0.04, Chi.square/DF=1.68, SRMR=0.10], indicating appropriateness of using this same 12-factor model in the DS cohort. These results followed Bartlett’s correction for sample size (see **Supplementary Materials** for R-function developed for that purpose).

**Table 2:** Internal consistency reliability of ND-PROM constructs across DS and ASD groups.

ND-PROM Domains	Cronbach’s Alpha DS/ASD/DS+ASD	McDonald’s Ω DS/ASD/DS+ASD
F1: Expressive Language	0.925/0.932/0.923	0.932/0.937/0.924
F2: Receptive Language	0.769/0.856/0.835	0.780/0.871/0.723
F3: Nonverbal Communication	0.803/0.835/0.694	0.816/0.851/0.723
F4: Social Emotional Understanding	0.830/0.883/0.899	0.836/0.885/0.906
F5: Social Interaction	0.871/0.907/0.889	0.865/0.906/0.893
F6: Independent Play	0.734/0.794/N.E.	0.795/0.820/N.E.
F7: Adaptive Skills/Toileting	0.772/0.632/0.752	0.868/0.821/0.797
F8: Restricted and Repetitive Behaviors and Interests	0.862/0.852/0.842	0.874/0.863/0.876
F9: Sensory Processes	0.578/0.632/0.599	0.564/0.592/0.538
F10: Challenging Behaviors	0.719/0.693/0.650	0.774/0.714/0.640
F11: Impulse/ADHD	0.773/0.701/0.635	0.786/0.730/0.682
F12: Mental Health	0.735/0.768/0.747	0.760/0.793/N.E.

Internal Consistency Reliability measures for Down syndrome (DS), and autism spectrum disorder (ASD) groups for each of the 12 domains of the ND-PROM. N.E., Not estimable; ADHD, attention deficit/hyperactivity disorder.

### 3.3 Measurement invariance across DS, ASD, and DS+ASD groups

**Table 3** displays results from the “exact” measurement invariance protocol when testing the 12 factor simple structure across the three groups. As expected, the chi-square statistical tests were significant for all three models, configural/metric/scalar with increased numbers of constraints. However, of interest were the comparisons between (a) configural and metric models, and (b) metric and scalar models. As shown in **Table 3**, neither the equivalence of factor loadings nor the equivalence of thresholds were supported when contrasting the DS and ASD samples. Consequently, the alignment procedure outlined above, was implemented to target partial measurement invariance so that tests of latent means would be possible.

**Table 3:** Measurement invariance across DS, ASD, and DS+ASD groups using an exact-fit protocol.

Model	Npar	Chi-square	D.F.	P-value
1.Configural	690	39812.99	12357	<0.001***
2.Metric	609	40290.17	12519	<0.001***
3.Scalar	528	43370.94	12681	<0.001***
4.Metric vs Configural	**-**	477.181	162^a^	<0.001***
5.Scalar vs Metric	**-**	3080.77	162^a^	<0.001***

Exact fit protocol for Down syndrome (DS), autism spectrum disorder (ASD), and dual diagnosis DS and ASD (DS+ASD) groups. *** denotes significance at p<0.001. Npar, Number of Freely Estimated Parameters; D.F., degrees of freedom. ^a^Indicates diffference in degrees of freedom across competing models.

Following alignment (33), results indicated that all but two of the factor loadings and all but eight of the intercepts were equivalent between the DS, ASD, and DS+ASD groups. Thus, the model was able to converge on a simplicity function where estimates of factor loadings and intercepts were largely invariant between groups. **Table 4** displays factor loadings and intercepts between groups and the decision of equivalence based on alignment. The amount of significant and non-invariant parameters was equal to 5.4%, much less than the 25% guideline and close to the nominal level of significance on the number of significant tests due to chance.

**Table 4:** Standardized factor loadings from a confirmatory factor analysis model for the 12 latent variables of the ND-PROM across DS, ASD and DS+ASD groups.

Factor	Items	DS Factor Loading	ASD Factor Loading	DS+ASD Factor Loading	Alignment of Item Loadings	Alignment of Item Intercepts
**Expressive Language**	Indicates yes/no	0.682	0.726	0.639	Yes	Yes
	Uses names of objects	0.813	0.817	0.755	Yes	Yes
	Requests/asks for things	0.791	0.736	0.698	Yes	Yes
	Makes comments	0.882	0.881	0.867	Yes	Yes
	Tells others what to do	0.812	0.840	0.749	Yes	Yes
	Asks “Why” questions	0.807	0.819	0.744	Yes	**No**
	Tells you about an event that happened in the past	0.845	0.839	0.766	Yes	Yes
	Has conversations	0.843	0.827	0.674	**No**	Yes
	Communicates spontaneously (initiates)	0.687	0.748	0.783	Yes	Yes
	Pronounces words correctly	0.693	0.747	0.554	Yes	**No**
**Receptive Language**	Understands when told Yes/No	0.667	0.613	0.646	Yes	Yes
	Understands 1 step directions	0.838	0.836	0.900	Yes	Yes
	Understands 2 step directions	0.863	0.893	0.785	Yes	Yes
	Understands if/then	0.769	0.870	0.808	Yes	Yes
	Understands non-literal language	0.558	0.630	0.537	Yes	Yes
	Responds when name is called	0.539	0.564	0.499	Yes	Yes
**Nonverbal Communication**	Points to indicate wants	0.734	0.688	0.608	Yes	Yes
	Points to share interest when not requesting	0.807	0.798	0.780	Yes	Yes
	Gestures	0.674	0.780	0.632	Yes	Yes
	Makes appropriate eye contact	0.458	0.463	0.345	Yes	Yes
	Uses facial expressions to show feeling	0.443	0.599	0.150	Yes	Yes
	Combines eye contact, gestures, facial expressions appropriately	0.623	0.763	0.496	Yes	Yes
**Social Emotional Understanding**	Distinguishes friendly teasing from bullying	0.635	0.710	0.564	Yes	Yes
	Recognizes emotions of others	0.579	0.660	0.717	Yes	Yes
	Demonstrates sportsmanship	0.684	0.718	0.617	Yes	Yes
	Identifies own feelings	0.721	0.660	0.602	Yes	Yes
	Understands others may have different point of view	0.744	0.771	0.517	Yes	Yes
	Shows remorse (being sorry)	0.713	0.744	0.750	Yes	Yes
	Handles criticism well	0.659	0.645	0.594	Yes	**No**
	Offers comfort to others	0.680	0.681	0.808	Yes	Yes
**Social Interaction**	Appropriately gets someone’s attention to start/end interaction	0.599	0.633	0.655	Yes	Yes
	Understands personal space	0.473	0.622	0.683	Yes	**No**
	Seems interested in interacting with children he/she knows	0.693	0.677	0.250	Yes	Yes
	Responds appropriately to greetings from children he/she knows	0.707	0.746	0.551	Yes	Yes
	Plays with classmate with help	0.555	0.591	0.497	Yes	Yes
	Plays with classmate without help	0.832	0.789	0.692	Yes	Yes
	Plays in group of classmates without help	0.813	0.769	0.600	Yes	Yes
	Imitates or copies others to learn	0.603	0.541	0.610	Yes	Yes
	Plays simple social games	0.613	0.671	0.377	Yes	Yes
	Plays cooperative games/taking turns and following rules	0.545	0.668	0.728	Yes	Yes
	Attempts to contact familiar children outside of school	0.525	0.673	0.122	Yes	Yes
	Understands social relationships	0.519	0.712	0.745	Yes	Yes
**Independent Play**	Engages in simple pretend play	0.458	0.654	0.714	Yes	Yes
	Acts out scene (scripted play)	0.784	0.735	0.799	Yes	Yes
	Pretends to be superhero or other character	0.894	0.809	0.801	Yes	Yes
**Adaptive Skills/Toileting**	Potty trained day	0.839	0.770	0.710	Yes	Yes
	Cleans/wipes	0.889	0.877	0.756	Yes	Yes
	Dresses independently	0.854	0.925	0.857	Yes	Yes
	Smears/plays with stool/urine(R)	0.261	0.303	0.480	**No**	Yes
	Toilets inappropriate places(R)	0.156	0.164	0.181	Yes	Yes
**Restricted and Repetitive Behaviors and Interests**	Focuses on unusual interests that interfere	0.492	0.608	0.326	Yes	Yes
	Focuses on intense interests that interfere	0.522	0.582	0.473	Yes	Yes
	Repetitive movements	0.492	0.346	0.617	Yes	Yes
	Simple repetitive activities	0.536	0.558	0.523	Yes	Yes
	Focuses on parts of objects	0.467	0.607	0.472	Yes	Yes
	Compulsions/rituals	0.664	0.567	0.645	Yes	Yes
	Avoids/upset about new places/people	0.646	0.582	0.559	Yes	Yes
	Easily upset with changes in routine	0.753	0.656	0.614	Yes	Yes
	Difficulty with transition	0.683	0.632	0.576	Yes	Yes
	Needs you to change your behavior to avoid becoming upset	0.710	0.593	0.500	Yes	Yes
	Speaks in unusual tone of voice	0.496	0.440	0.426	Yes	Yes
	Repeats meaningless sounds	0.400	0.416	0.404	Yes	Yes
	Echoes other people	0.348	0.396	0.485	Yes	Yes
	Repeats phrases from TV/movies	0.477	0.446	0.620	Yes	Yes
	Perseverates or gets stuck	0.570	0.502	0.769	Yes	Yes
**Sensory Processes**	Peers out of corner of eyes	0.400	0.496	0.617	Yes	Yes
	Craves deep pressure	0.526	0.486	0.655	Yes	Yes
	Upset by noises	0.411	0.373	0.343	Yes	Yes
	Puts things into mouth that are not food	0.352	0.467	-0.025	Yes	Yes
	Avoids touching certain things	0.493	0.547	0.184	Yes	Yes
	High tolerance for pain	0.337	0.242	0.606	Yes	Yes
	Holds or packs food in mouth	0.333	0.225	-0.049	Yes	Yes
	Eats limited variety of foods	0.280	0.442	0.385	Yes	Yes
**Challenging Behaviors**	Physically aggressive toward self	0.564	0.455	0.613	Yes	**No**
	Physically aggressive towards others	0.743	0.594	0.687	Yes	Yes
	Expresses thoughts of wanting to hurt others	0.383	0.435	0.148	Yes	Yes
	Destroys or breaks things when upset	0.767	0.598	0.637	Yes	Yes
	Temper tantrums or meltdowns	0.693	0.774	0.803	Yes	**No**
	Interrupts when others are speaking	0.397	0.360	0.239	Yes	Yes
**Impulse/ADHD**	Runs away	0.592	0.332	0.302	Yes	Yes
	Easily distracted, difficulty paying attention	0.615	0.628	0.322	Yes	Yes
	Hyperactive	0.705	0.770	0.725	Yes	Yes
	Impulsive, acts without thinking	0.855	0.787	0.800	Yes	Yes
**Mental Health**	Expresses self-harm or suicide	0.215	0.445	0.224	Yes	Yes
	Victim of bullying	0.363	0.528	0.215	Yes	Yes
	Worries too much	0.516	0.646	0.469	Yes	Yes
	Picks at skin or nails	0.380	0.476	0.445	Yes	Yes
	Seems sad	0.553	0.708	0.456	Yes	Yes
	Easily frustrated	0.710	0.716	0.789	Yes	**No**
	Sudden changes in mood	0.791	0.712	0.627	Yes	**No**
	Sees things not there	0.345	0.208	0.074	Yes	Yes
	Hears things not there	0.267	0.195	0.195	Yes	Yes
	Decreased or flattened emotions	0.378	0.403	0.495	Yes	Yes

Confirmatory factor analysis standardized factor loadings for Down syndrome (DS), autism spectrum disorder (ASD), and dual diagnosis DS and ASD (DS+ASD) groups on the ND-PROM. Estimates are standardized.

### 3.4 Latent mean differences across DS, ASD, and DS+ASD groups

**Figure 1:**
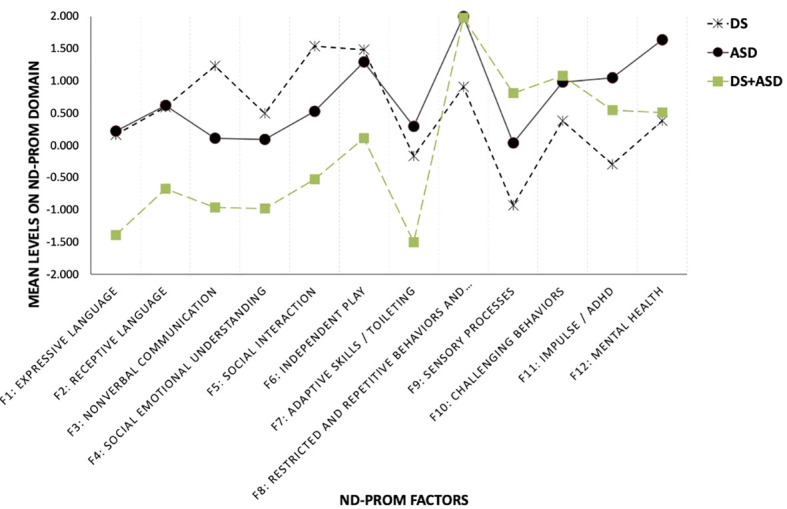
Latent mean differences between DS, ASD and DS+ASD groups on ND-PROM factors. Mean differences for each of the 12 factors of the Neurodevelopmental Parent Report for Outcome Montioring (ND-PROM) survey are shown for three groups: Down syndrome (DS; n=385), autism spectrum disorder (ASD; n=246), and a dual diagnosis of DS and ASD (DS+ASD; n=53).

**Table 5** and **Figure 1** display latent means and between group comparisons using both inferential statistical criteria and effect size indicators. As shown in **Table 5**, there were significant differences between the DS and ASD groups on nonverbal communication and social interaction, with the DS group having significantly higher means (nonverbal communication difference=1.021 SD; social interaction difference=0.933 SD). Higher means in these areas are indicative of higher skill levels. Similarly significantly lower scores in the ASD were observed in RRBs (-1.078 SD), sensory processes (-0.881 SD), challenging behaviors (-0.614 SD), impulse/ADHD (-1.376 SD), and mental health (-1.050 SD). Last, there were significant differences between the DS and ASD groups in adaptive skills/toileting (-0.458 SD); with the DS group having significantly lower mean levels compared to the ASD group.

**Table 5:** Latent mean differences between DS, ASD, and DS+ASD groups using standardized point estimates.

DS-Prom Latent Factor	Mean DS	Mean ASD	Mean DS+ASD	Latent *d* *DS vs. ASD*	Latent *d* *DS vs. DS+ASD*	Latent *d* *ASD vs. DS+ASD*
F1: Expressive Language	0.167	0.218	-1.390	-0.050	1.558^†^*	1.579^†^*
F2: Receptive Language	0.595	0.621	-0.677	-0.026	1.262^†^*	1.261^†^*
F3: Nonverbal Communication	1.230	0.108	-0.963	1.021^†^*	2.216^†^*	0.904^†^*
F4: Social Emotional Understanding	0.497	0.094	-0.982	0.393	1.489^†^*	1.032^†^*
F5: Social Interaction	1.536	0.526	-0.525	0.933^†^*	2.079^†^*	0.909^†^*
F6: Independent Play	1.484	1.293	0.113	0.196	1.390^†^*	1.275^†^*
F7: Adaptive Skills/Toileting	-0.168	0.291	-1.503	-0.458	1.366^†^*	1.852^†^*
F8: Restricted and Repetitive Behaviors and Interests	0.907	2.000	1.979	-1.078^†^*	-1.064^†^*	0.020
F9: Sensory Processes	-0.933	0.034	0.806	-0.881^†^*	-1.672^†^*	-0.620^†^
F10: Challenging Behaviors	0.377	0.983	1.076	-0.614^†^*	-0.699^†^	-0.096
F11: Impulse/ADHD	-0.293	1.048	0.545	-1.376^†^*	-0.841^†^*	0.535^†^*
F12: Mental Health	0.381	1.636	0.506	-1.050^†^*	-0.128	0.833^†^

Differences in latent means between Down syndrome (DS), autism spectrum disorder (ASD), and dual diagnosis DS and ASD (DS+ASD) groups on the ND-PROM. *denotes significance at p<0.05. ^†^denotes differences greater than medium (i.e., 0.5 standard deviations) based on Cohen (1992).

When contrasting the DS to the DS+ASD groups, results indicated significantly higher functioning for the DS group in expressive language (1.558 SD), receptive language (1.262 SD), nonverbal communication (2.216 SD), social communication (1.489 SD), social interaction (2.079 SD), independent play (1.390 SD), and adaptive skills (1.103 SD). A significantly lower functioning of the combined group was observed in restricted and repetitive behaviors and interests (-1.064 SD), sensory processes (-1.672 SD), challenging behaviors (-0.699 SD), and impulse/ADHD (-0.841 SD). There were no differences between the DS and combined DS+ASD groups for the mental health domain (-0.128 SD).

Comparisons between the ASD and the DS+ASD groups indicated that the ASD group was significantly higher in expressive language (1.579 SD), receptive language (1.261 SD), nonverbal communication (0.904 SD), social communication (1.032 SD), social interaction (0.909 SD), independent play (1.275 SD), and adaptive skills (1.593 SD), all areas where higher means indicate higher skill or functioning levels. However, the ASD group also had a higher mean for impulse/ADHD (0.535 SD). Significantly lower functioning was observed for the combined group on sensory processes (-0.620 SD).

## 4 Discussion

The present study evaluated the ND-PROM as a clinical monitoring tool to assess skills and behaviors in the DS population. The study evaluates internal structure validity of the ND-PROM with measurement invariance across DS, ASD, and DS+ASD samples. Our findings support the existence of the 12 clinically derived factors in the DS population: Expressive Language, Receptive Language, Nonverbal Communication, Social Emotional Understanding, Social Interaction, Independent Play, Adaptive skills/Toileting, Restricted and Repetitive Behaviors and Interests, Sensory Processes, Challenging Behaviors, Impulse/ADHD, and Mental Health. This 12-factor model was previously confirmed through confirmatory factor analysis in the ASD population, and here we have shown that the ND-PROM tool works equally well and can capture the range of distinct and independent areas of developmental and behavioral functioning present in those with DS. Assessment of measurement invariance across DS, ASD, and DS+ASD groups showed that the ND-PROM was able to psychometrically distinguish between these three groups, indicating the specificity of the tool in elucidating different patterns of development and behaviors between children with DS, ASD, and DS+ASD.

Assessment of latent mean differences between DS and ASD revealed differences in patterns of development, and skills, and behaviors in those with DS compared to those with ASD. Expressive and receptive language, independent play skills, sensory processes, and challenging behaviors were similar in the sample populations of ASD and DS in the study. However, those with DS were found to have higher abilities in nonverbal communication, social emotional understanding, social interaction, and fewer reported restricted and repetitive behaviors and interests, impulsivity or ADHD symptoms, and mental health concerns compared to children with ASD in this study. These findings are largely in line with prior reports describing profiles for children with DS. Children with DS have been described to have relative strengths in nonverbal communication and social skills (2, 7, 35, 36), whereas these are defining core areas of impairment for children with ASD (22). Adaptive skills have previously been reported to be higher for children with DS compared to those with ASD (37, 38), however in this study focused on toileting skills, this pattern was not found. Additionally, while challenging behaviors and mental health concerns, as well as restricted and repetitive behaviors, and impulse control/ADHD symptoms are commonly reported in DS, they may occur less than in other populations of children with ID (4, 39).

Compared to the DS-only group, the DS+ASD group shows areas of concern in language and communication domains, as well as in social interaction, independent play, and adaptive skills. The combined group also had more issues with restricted and repetitive behaviors and interests, sensory processing, and challenging behaviors. This is consistent with previous research showing vulnerabilities in children and adolecents with co-occurring DS and ASD (12, 40, 41). When comparing to the ASD-only group, the DS+ASD group had decreased functioning in communication and language, including social communication and social interaction, as well as adaptive skills. This differs from previous research which showed fewer issues with social interaction in a combined DS+ASD group compared to an ASD-only group (42). Interestingly, the ASD-only group had higher levels of impulse/ADHD symptomatology reported.

We recognize several limitations of the current work. The study population included primarily White, non-Hispanic respondents, thus may not be representative. However, the ND-PROM is now available in additional languages, thus future analyses can include a more diverse population. Though previously parents reported that use of the ND-PROM had a positive impact on their child’s care (20), the length of the ND-PROM may be a limitation for its widespread application, especially in primary care clinics or other, non-tertiary care environments. In this study, the DS and ASD cohorts were not matched by cognitive level, thus more pronounced group differences may have been observed if the DS group were directly compared to a group with ASD and Intellectual Disability. Additionally, this study did not assess the stability of responses across repeated tests or the sensitivity of the measure to change over time, and therefore it is not clear how clinically meaningful changes in function might be represented on the ND-PROM. Additionally, though convergent validity was previously assessed in the ASD propulation (20), it was not repeated in the DS population as a part of this study. Finally, the ND-PROM responses were collected as part of clinical care visits to a specialty clinic within a tertiary pediatric care center. Therefore, patients included in both the DS and ASD samples might be more severely impacted than their peers in the general population, hence their desire to seek out specailized care. However, this scale was designed to facilitate clinical visits in the medical setting and this study has shown that it is useable within that setting. A last cautionary note relates to the relatively low internal consistency reliability estimates for some of the ND-PROM scales. In these instances we suggest caution about using the scores from individual domains for diagnostic and classification purposes. Instead future studies may consider revising the content of some of these scales so that levels of internal consistency reliability will increase.

Future directions will explore the use of the ND-PROM longitudinally to assess the ability to capture change over time, which will have implications when assessing response to intervention. Additionally, as the ND-PROM is now available in additional languages, future studies will include a more diverse population. Subsequent studies may also include an examination of a Computerized Adaptive Testing (CAT) framework using the computerized version of the ND-PROM, such that larger item pools can be created and an adaptive algorithm can be implemented to assess competency in each domain through defining a minimum tolerated error of measurement. CAT methodologies engage approximately 10-15% of the total number of items and thus, the gains in time, efficiency, with no sacrifice to the validity of the measure would enable increased use and scalability of the ND-PROM.

The ND-PROM is a clinically useful tool for assessing children and adolescents with DS, ASD, and DS+ASD, which captures a range of distinct and independent areas of developmental and behavioral functioning between and among these three groups.

## Data availability statement

The datasets presented in this article are not readily available because the data contain protected Personal Health Information. Requests to access the datasets should be directed to NB, nicole.baumer@childrens.harvard.edu.

## Ethics statement

The studies involving humans were approved by Boston Children's Hospital Institutional Review Board. The studies were conducted in accordance with the local legislation and institutional requirements. The ethics committee/institutional review board waived the requirement of written informed consent for participation from the participants or the participants' legal guardians/next of kin because a waiver was issued; information included retrospectively analyzed data that was collected for clinical purposes.

## Author contributions

NB: Conceptualization, Formal Analysis, Investigation, Writing – original draft, Writing – review & editing. KP: Conceptualization, Project administration, Visualization, Writing – review & editing. BZ: Conceptualization, Methodology, Supervision, Validation, Writing – review & editing. GS: Conceptualization, Formal Analysis, Investigation, Methodology, Software, Visualization, Writing – original draft.
