# Supplementary material for: Validation of factor structure of the neurodevelopmental parent report for outcome monitoring in down syndrome: confirmatory factor analysis
Source: Front Psychiatry. 2024 Mar 5;15:1293937. doi: 10.3389/fpsyt.2024.1293937 (PMC10948425; doi:10.3389/fpsyt.2024.1293937)
Supplement: Supplementary file 1 [file DataSheet_1.docx]

Validation of Factor Structure of the Neurodevelopmental Parent Report for Outcome Monitoring (ND-PROM) in Down Syndrome: Confirmatory factor analysis

Supplementary Material

# Supplementary Figures

**Supplementary Figure 1.** R-Function developed to assess the effects of sample size on omnibus chi-square test and fit indices in confirmatory factor analysis. RMSEA=root mean square error of approximation; DF=degrees of freedom; NFI=normed fit index; IFI=incremental fit index; TLI=Tucker-Lewis Index; CFI=comparative fit index; GFI=goodness of fit index; AGFI=adjusted goodness of fit index; gamma-h=gamma hat index; RNI=relative noncentricity index ; NCI=non-centrality index. cfagofi1(12, 93, 384, 7708.876, 4119, 31367.221,4278, FALSE, FALSE, FALSE)
